# The distribution of *Blastocystis* subtypes in isolates from Qatar

**DOI:** 10.1186/s13071-015-1071-3

**Published:** 2015-09-17

**Authors:** Marawan Abu-Madi, Mahmoud Aly, Jerzy M. Behnke, C. Graham Clark, Hanan Balkhy

**Affiliations:** Department of Health Sciences, College of Arts and Science, Biomedical Research Center, Qatar University, P.O. Box 2713, Doha, Qatar; King Abdullah International Medical Research Center, National Guard Health Affairs, Mail Code: 2216, P.O. Box 22490, Riyadh, 11426 KSA; King Saud bin Abdulaziz University for Health Sciences, Riyadh, Saudi Arabia; School of Life Sciences, University of Nottingham, University Park, Nottingham, United Kingdom NG7 2RD; Faculty of Infectious and Tropical Diseases, London School of Hygiene and Tropical Medicine, Keppel Street, WC1E 7HT London, United Kingdom

**Keywords:** *Blastocystis*, Real time-PCR, Subtype, Small subunit ribosomal DNA, Prevalence, Genotyping, Phylogenetic analysis, Qatar

## Abstract

**Background:**

*Blastocystis* is a common single-celled intestinal parasite of humans and other animals comprising at least 17 genetically distinct small subunit ribosomal RNA lineages (subtypes (STs)), nine of which have been found in humans. The geographic distribution of *Blastocystis* subtypes is variable, but the subtypes present in Qatar are at present unknown.

**Methods:**

Stool samples were collected from randomly selected, apparently healthy subjects arriving in Qatar for the first time. *Blastocystis* subtypes were determined by sequencing of the small subunit rRNA gene (SSU rDNA) PCR products. Phylogenetic analyses were done using Maximum Composite Likelihood method.

**Results:**

71.1 % of samples were positive for *Blastocystis* infection based on PCR-detection methodology compared to only 6.9 % by microscopy. Prevalence of *Blastocystis* did not differ between the sexes nor between age classes. However, there was a regional difference in prevalence with subjects arriving from Africa showing the highest (87.6 %), those from Western Asia intermediate (68.6 %) and from Eastern Asia the lowest prevalence (67.6 %). Genetic analysis detected only three STs. ST3 was the most common (69.3 %) and ST2 was the rarest (3.5 %), while ST1 had a prevalence of 27.2 %. ST2 showed a regional variation, being absent from the 64 Western Asian *Blastocystis-*positive subjects. Both ST1 and ST3 showed significant differences in prevalence between the sexes.

**Conclusions:**

This is the first report exploring the distribution of *Blastocystis* subtypes in our region. We recommend that stool screening via microscopy for the presence of *Blastocystis* should be abandoned since it is extremely insensitive. In future, the prevalence of *Blastocystis* infections should be based on PCR methodology and we predict that in the years ahead diagnostic PCR will become the tool of choice. More work is needed to identify the full range of *Blastocystis* subtypes that circulate in our region.

## Background

*Blastocystis* is a single-celled intestinal protist, taxonomically placed within the Stramenopiles, which colonizes an estimated 1,000,000,000 people globally and a variety of animal species [1]. Although *Blastocystis* is also detected in asymptomatic humans [2], some studies link this organism with intestinal and extra-intestinal disease [3, 4]. To date, despite *Blastocystis* being the most frequently isolated protist from diarrheal patients in the developed world, a causal link has not yet been established conclusively [5]. *Blastocystis* is genetically diverse and based on small subunit rRNA gene analysis (SSU rDNA), at least 17 subtypes (STs) have been identified in humans, other mammals, and birds. Among these subtypes, ST1-ST4 collectively account for 90 % of human carriage [6], while the ST5-ST9 account for the remaining 10 %. To date ST9 has only been isolated from humans [7]. To better understand the genetic diversity and determine the prevalence of *Blastocystis* subtypes around the world, further research is required [8, 9]. The introduction of molecular screening assays such as real-time polymerase chain reaction (RT-PCR) has demonstrated that the prevalence of *Blastocystis* is much higher than previously reported on the basis of detection by conventional microscopy, in both developed [2, 9, 10] and developing [6] countries. Studying the genetic diversity of *Blastocystis* in different hosts, age groups and genders, and in different regions of the world, is essential to further our knowledge of the epidemiology and clinical relevance of this organism.

In the past decade, Qatar has seen a fast-paced transformation in the standards of living of its citizens. This tremendous growth has spawned building and modernization programs. There has been a large influx of migrant workers into Doha to complete very ambitious and large-scale construction projects. These workers mostly originate from states in the Middle-East, Asia and Africa, the latter two being regions where intestinal parasites are particularly common [11, 12]. Economic growth has been accompanied by rapidly expanding domestic services in the city, which are also dependent on an immigrant labour force. Improvements in the infrastructure have not kept pace with this transformation, particularly the housing and sanitation available for workers. The immigration policy in Qatar applies a quota system to control the number of workers entering Qatar each year according to their country of origin. This creates a dynamic population of workers that changes from year to year and therefore requires continuous monitoring. On arrival in Qatar and before they can obtain work permits, all new immigrants are obliged to report to the Medical Commission for thorough medical inspections but fecal examinations of all immigrant workers seeking employment in the food industries and/or as housemaids were abandoned following the introduction of compulsory treatment with albendazole, which is effective mainly against helminths and not most protozoa (although it does have activity against *Giardia* [13]). No specific treatment is given for protozoan infections, so in contrast to helminth infections these are not eradicated from infected immigrants on arrival in Qatar. Once a worker has been issued a work permit they are then not obliged to undergo further health inspections unless they work in the food industry, in which case annual re-examination is mandatory. As foreign workers form an integral part of the food industry in Qatar, it is imperative to determine their status as a potential reservoir and source of infection for enteric pathogens. The same is true for housemaids, who not only handle the food served to families, but also infants and children, who are generally more susceptible to infection than adults.

As prevention is far more effective in the long-term than cure, the identification of carriers and facilitation of the medical management of individuals who shed these pathogens can be improved through the application of sensitive screening methods, thereby minimizing the risk of infection spreading to other sectors of the community. In this paper we report on the implementation of a molecular screening assay (MSA) based on real-time PCR for the detection and subtyping of *Blastocystis* in samples collected from migrant workers newly arriving in Qatar, and we compare the detection rate with that achieved by conventional microscopy. We examine prevalence data for evidence of regional variation, while controlling for age and sex effects, and in a subset of the data we test whether the prevalence of the different STs of *Blastocystis* differs between regions of origin of the carriers or is influenced by host intrinsic factors, sex and age.

## Methods

### Sample collection and DNA extraction

We obtained stool samples from 608 randomly selected immigrant workers (including food handlers, construction workers, and housemaids), arriving for the first time in Qatar, and undergoing mandatory health checks at the Medical Commission during their application for work permits. Samples were collected in individually labelled, standard sterile disposable containers and were immediately stored on ice until processed for DNA extraction and microscopy.

Samples were aliquoted for DNA extraction alongside conventional stool examination by microscopy. Microscopic examination was carried out as described by Abu-Madi and others [14]. DNA was extracted using the QIAamp DNA stool minikit (Qiagen, Hilden, Germany) according to the manufacturer’s instructions. Briefly, samples were weighed, homogenized in lysis buffer, and incubated at 95 °C for 5 min to ensure lysis of the targeted protozoa. After centrifugation, the DNA in the supernatant was purified using a silica column supplied with the kit. The quantity and quality of the DNA was determined using spectrophotometry (Nanodrop, ThermoScientific) and gel electrophoresis.

### Ethical approval

Ethical approval for this project was obtained from the Medical Research Centre and the Research Committee at Hamad Medical Corporation, Qatar (Research protocol # 11110/11).

### RT-PCR amplification and sequencing

To detect the presence of *Blastocystis* in the DNA extracted from the samples described above, well-studied primers targeting the SSU rDNA region were used [10, 15, 16]. This region covers approximately 190 bp that are highly specific for the SSU rDNAs of *Blastocystis* STs1-9, but can also distinguish each ST. This approach has been chosen over other possible approaches as the primer set used is of diagnostic quality yet at the same time it amplifies a product that will allow for subtyping once it is sequenced [15, 16]. DNA extracted from samples that were positive for *Blastocystis* based on light microscopy was used as a positive control. The reaction mixture contained a final concentration of 0.3 μM of the primer set and 0.125 μM probe. Samples were processed using an ABI 7500 instrument. The reaction conditions were set as following: 95 °C for 5 min, and 50 cycles of denaturation at 95 °C for 15 s followed by annealing and extension at 60 °C for 1 min.

Purified PCR products were sequenced using a BigDye® Terminator v3.1 Cycle Sequencing Kit (Applied Biosystems™ Austin, TX, USA). Thermal cycling was 96 °C for 1 min, 40 cycles of (96 °C for 10 min, 50 °C for 5 s, 60 °C for 4 min) using either *Blastocystis* Fwd S1, GGTCCGGTGAACACTTTGGATTT or *Blastocystis* RvsS2, CCTACGGAAACCTTGTTACGACTTCA. Products were purified using XTerminator™ and SAM™ solutions (Applied Biosystems™ Foster City, CA, USA) and sequenced on a 3730xl DNA Analyzer (Applied Biosystems™, Hitachi, Tokyo, Japan).

### Genotyping analysis

To identify *Blastocystis* subtypes*,* both strands of the amplicons were sequenced and were compared with all the SSU rRNA gene sequences available from the National Centre for Biotechnology Information (NCBI) database using the BLAST program. SSU rDNA subtypes were identified by determining the closest similarity match against all known *Blastocystis* STs. All of our sequences have been deposited in GenBank under Accession numbers KM438204-30.

### Statistical analysis

Prevalence data are presented as percentages with 95 % confidence limits (95 % CL), calculated with bespoke software based on the tables of Rohlf and Sokal [17]. Analysis comprised three stages. In the first we compared detection of *Blastocystis* using the PCR method with conventional detection by routine microscopy.

In the second step, we analyzed the factors affecting prevalence of *Blastocystis*, based on detection of infected subjects by PCR. For this analysis we used maximum likelihood techniques based on log linear analysis of contingency tables in the software package IBM SPSS Statistics Version 21 (IBM Corporation). Initially, full factorial models were fitted, incorporating as factors region of origin of subjects (REGION - 3 levels), sex of subjects (SEX, 2 levels, male and female), and age of subjects (AGE, 3 levels). The subjects (*n* = 608) originated from 18 countries, but for statistical analysis were assigned to three regions. Region 1, Africa comprised subjects from Cameroon (*n* = 1), Egypt (2), Eritrea (3), Ethiopia (34), Ghana (3), Kenya (27), Nigeria (15), Sudan (2), Chad (1) and Uganda (1). Region 2, Eastern Asia comprised subjects from Indonesia (119), Philippines (65), Thailand (2) and Vietnam (2). Region 3, Western Asia, comprised subjects from Bangladesh (71), India (117), Nepal (62) and Sri Lanka (81). Subjects were allocated to three age classes (age class 1 = 18–29 years old, age class 2 = 30–39 years old and age class 3 = 40–58 years old). The presence / absence of *Blastocystis* (INFECTION) was fitted as a binary factor. These explanatory factors were fitted initially to all models that were evaluated. For each level of analysis in turn, beginning with the most complex model, involving all possible main effects and interactions, those combinations that did not contribute significantly to explaining variation in the data were eliminated in a stepwise fashion beginning with the highest level interaction (backward selection procedure). A minimum sufficient model was then obtained, for which the likelihood ratio of *χ*2 was not significant, indicating that the model was sufficient to explain the data. The importance of each term (i.e. interactions involving infection) in the final model was assessed by the probability that its exclusion would alter the model significantly and these values relating to interactions that included INFECTION are given in the text.

In stage three, analysis of the factors affecting the prevalence of genotyped *Blastocystis* isolates was conducted on the smaller data-set of sequenced isolates. The subtype identification of a subset of 132 randomly selected isolates was achieved by sequencing the SSU rDNA. Some isolates (*n* = 18) were problematic and not available for analysis because they could not be subtyped successfully or were associated with missing data. However, 114 isolates were completely subtyped and analysed for the current study. The successfully subtyped isolates originated from donors ranging in age from 20 years to a maximum of 56 years, with an average of 31.9 ± 0.75 (median of 30.0) years. Fifty three subjects were males (mean age = 31.2 ± 1.13 and 61 females (mean age = 32.5 ± 0.99), and there was no significant difference between the ages of the two sexes (*z* = 1.055*, P* = 0.29). The approach adopted for analysis was similar to that in stage two of the analysis. Again subjects were allocated to the same three regions and age classes. Three models were fitted, one each for *Blastocystis* ST1, ST2 and ST3, and in each case with AGE, SEX and REGION as explanatory factors and presence/absence of the subtype (INFECTION) as a binary factor. Reduction to minimum sufficient models followed the same procedure as that explained above in stage two of the analysis.

Throughout our cut-off for statistical significance was *P* = 0.05.

## Results

### Prevalence of *Blastocystis* based on PCR method of detection

Of the 608 samples tested by PCR for the presence of *Blastocystis*, 432 were positive and 176 negative giving an overall prevalence of 71.1 %. In contrast, microscopy only identified 42 positives (6.9 % prevalence). Thirty seven samples were identified as positive and 171 as negative by both methods. However, five samples were recorded as positive by microscopy but not detected as such by PCR.

### Factors affecting the prevalence of *Blastocystis* based on detection by PCR

Analysis of the prevalence of *Blastocystis* based on PCR revealed that neither host sex nor age affected prevalence significantly (Table 1). However, there was a highly significant effect of the region from which subjects originated (REGION x INFECTION, *χ*^2^_2_ = 16.1, *p* <0.001). Infections with *Blastocystis* were most common among subjects from Africa and least common among those from Eastern Asia, and the difference in prevalence between these regions was 20.0 %.

**Table 1:** Factors affecting prevalence of *Blastocystis*, based on detection by PCR

Factor	Number	Prevalence (%)	95 % CL
Host sex			
Males	280	70.7	65.85–75.17
Females	328	71.3	66.10–76.10
Host age			
Age class 1 (18–29)	286	73.1	68.25–77.42
Age class 2 (30–39)	233	70.0	65.49–74.10
Age class 3 (40–58)	89	67.4	54.32–78.65
Region of origin			
Africa	89	87.6	76.54–94.08
Eastern Asia	188	67.6	57.95–75.86
Western Asia	331	68.6	63.23–73.49

### Subtype analysis and prevalence

Subtype analysis revealed that only three subtypes were present in the samples analyzed. ST1 was present in 31 samples (27.2 %), ST2 was found in four hosts (3.5 %), one from Nigeria and three from Indonesia, while 79 (69.3 %) samples had ST3. Interestingly, ST3 was found in samples isolated from subjects of all geographical regions included in the current study i.e. Africa, East and West Asia. Table 2 shows the occurrence of subtypes and strains among the 114 subjects by country of origin. When the data were subdivided by the 12 countries of origin of the subjects, some subsets became very small, and indeed there was only one subject each from Cameroon, Chad and Eritrea.

**Table 2:** The distribution of subtypes of *Blastocystis* among the 114 subjects by their countries of origin

Nationality	*n*	ST1	ST2	ST3
Africa	19			
Cameroon	1	1	0	0
Chad	1	1	0	0
Eritrea	1	1	0	0
Ethiopia	12	7	0	5
Kenya	2	0	0	2
Nigeria	2	0	1	1
East Asian	31			
Indonesia	19	6	3	10
Philippines	12	4	0	8
West Asia	64			
Bangladesh	12	3	0	9
India	20	3	0	17
Nepal	16	1	0	15
Sri Lanka	16	4	0	12
	114	31	4	79

### Factors affecting the prevalence of subtypes of *Blastocystis* in subsets of the subjects in the study

In order to enable statistical analysis, the subjects were allocated to three geographical regions (Tables 2 and 3). There was no regional effect on ST1 and ST3, but with age and host sex taken into account there was a significant effect of region of origin on ST2 (REGION x INFECTION, *χ*^2^_2_ = 7.1, *P* = 0.029), despite the low prevalence of this ST among the 114 samples. This subtype was not detected among the 64 W. Asian subjects (Table 2). Table 3 shows that ST3 appeared to be more common among W. Asian and ST1 among African subjects, but with host sex and age taken into account these differences in prevalence did not reach statistical significance (effect of REGION on ST1, *χ*^2^_2_ = 5.1, *P* = 0.078 and on ST3, *χ*^2^_2_ = 5.9, *P* = 0.052).

**Table 3:** Prevalence of subtypes of *Blastocystis* among the three regions of origin of the subjects in the study

Region	*n*	Subtype prevalence as a percentage (95 % CL)		
		ST1	ST2	ST3
Africa	18	52.6 (31.18–74.29)	5.3 (0.27–25.70)	42.1 (22.19–65.51)
East Asia	28	32.3 (20.18–46.45)	9.7 (3.77–21.51)	58.1 (43.81–71.40)
West Asia	64	17.2 (10.19–27.20)	0 (0–4.80)	82.8 (72.80–89.81)

With region of origin and age taken into account, there were significant effects of host sex on the prevalence of ST1 (Table 4; SEX x INFECTION, *χ*^2^_1_ = 13.4, *p* <0.001) and ST3 (Table 4; SEX x INFECTION, *χ*^2^_1_ = 15.1, *p* <0.001). In the case of ST1 the bias was strongly in favour of female subjects with prevalence of this subtype being 3.6-fold more common among females. ST3, showed a bias in the opposite direction with prevalence among male subject being 1.6-fold higher compared with females.

**Table 4:** The influence of host sex and age on the prevalence of ST1 and ST3

Subset	ST1		ST3	
	%	95 % CL	%	95 % CL
Males	11.3	6.12–19.43	86.8	78.52–92.57
Females	41.0	30.62–51.87	54.1	43.19–64.47
Age class 1 (18–29)	30.0	18.80–43.96	66.0	52.02–78.23
Age class 2 (30–39)	27.3	14.46–44.25	68.2	51.16–82.03
Age class 3 (40–58)	20.0	7.14–42.35	80.0	57.65–92.86

With region of origin and host sex taken into account, there were no significant effects of host age, although as can be seen from Table 4, there appeared to be a steady decline in the prevalence of ST1 with increasing age and an increase with age in the case of ST3.

## Discussion

The results of our study show clearly that there is an enormous difference in the detection of *Blastocystis* in stool samples by microscopy vs PCR methodology. Based on PCR methodology we concluded that 71.1 % of the samples contained *Blastocystis* DNA, whilst conventional microscopy detected only 42 positive samples, giving a prevalence of just 6.9 %. Higher detection rates of intestinal protozoa, including *Blastocystis*, by PCR compared with conventional microscopy have also been recently reported in asymptomatic individuals from Brazil [18], where microscopy detected 10 infected subjects out of 126 (prevalence = 7.9 %) whereas PCR detected 43 infections in the same subjects (prevalence = 34.1 %). This difference in sensitivity of the two assays raises questions about the utility of continuing to employ conventional microscopy, when this method has such a high failure rate in detecting the presence of this parasite. In this study, as in our earlier work from Qatar, the stool samples were all screened by highly trained medical laboratory technicians and it is unlikely that any further improvement is possible in the accuracy of detection by microscopy. On this basis, we can confidently conclude that earlier estimates of the prevalence of *Blastocystis* in Qatar, which were all entirely based on microscopy [14], were very heavily underestimated. Clearly, diagnostic microscopy of *Blastocystis* infections is generally less sensitive than PCR-based methodology, resulting in marked underestimation of the true prevalence of *Blastocystis*.

Our data showed that there was no difference in prevalence between males and females both having similarly high values for prevalence, although others have found significant differences in prevalence between the sexes. Higher prevalence of *Blastocystis* in male compared with female subjects has been observed, for example, in Libya [19] and in China [20]. Participation in outdoor activities by the adult males, with the associated higher risk of contamination by the faecal-orally transmitted cysts of the parasite, has been proposed as the underlying explanation. However, there are examples also of bias in the opposite direction with higher prevalence among female subjects, as for example in Spain [21].

There was no difference in prevalence between the age classes in our data, although there was a consistent, if marginal, drift downwards with increasing age, which was not statistically significant. Some earlier studies concur with our results in failing to find any age effects on the prevalence of *Blastocystis*. For example, no significant associations were found between infections with any of the observed *Blastocystis* STs and age classes in Libya [22]. However, other studies have found significantly higher infection rates in adults compared with children, with the highest prevalence rate among asymptomatic young adults aged between 18 and 30 years [23]. Trends in the opposite direction, with a higher prevalence rate in children compared to adults, have also been reported, as for example in the Philippines [24]. In support of the latter, a recent study in Thailand has also reported a significant reduction in the *Blastocystis* infection with increasing age, the prevalence rate peaking in the younger children in the study [25]. These contrasting findings suggest that the distribution of *Blastocystis* infection among host populations shows spatial heterogeneity with respect to host age and sex, and these inconsistencies are most likely attributable to local factors such as the environmental conditions that influence locally the extent of, and the efficiency of, the faecal-oral route of transmission among host sectors of varying age, and between the two sexes [22].

The most prominent source of variation in prevalence of *Blastocystis* that we detected was in the region of origin of the subjects, with immigrants from Africa showing the highest prevalence and those from Eastern Asia, comprising Indonesia and the Philippines, the lowest. The prevalence of *Blastocystis* infection is known to vary from country to country, as well as within countries [6]. However, it has been reported that a higher prevalence occurs in developing countries than developed countries [26–28]. *Blastocystis* is transmitted through the faecal-oral route and among the most obvious risk factors are poor personal and community hygiene, culture, and lifestyle of a population. All of these vary from country to country, partially depending on the economic status and standard of living, but also on the geographical location and climatic factors [29].

Because we used a PCR-based methodology for detection and successfully sequenced the PCR products, we were also able to distinguish between different STs of *Blastocystis*. Our results revealed some interesting trends with respect to the influence of the region of origin on the prevalence of STs but the only ST that was shown statistically to vary in relation to where the carrier originated from was ST2, which was completely absent in the 64 subjects from W. Asia. It should be noted that only four samples in total were classified as this ST, and therefore we have to reserve judgement as to whether the conclusion of a regional influence on the prevalence of this ST is robust. Clearly further work must include a considerably larger sample size, to consolidate or refute this finding.

The high prevalence of ST3 in our study population, especially among immigrants from Western Asia, is consistent with other reports in the literature, as for example in Thailand [30], Egypt [31], Singapore [26], Turkey [32, 33], Germany [34], France [35], Malaysia [36], and Lebanon [37]. In a recent study of the distribution of *Blastocystis* subtypes in three African countries (Libya, Nigeria and Liberia) by Alfellani *et al*. [6], four subtypes were detected in the Libyan population with ST1 (50.0 %, 19/38) showing the highest prevalence, followed by ST3 (39.5 %, 15/38), ST2 (7.9 %, 3/38) and ST7 (2.6 %, 1/38). Other studies have also identified ST1 as dominant among the examined outpatients [19].

Perhaps the most interesting finding from our study, was the marked sex-difference in the prevalence of ST1 and ST3, with ST1 being far more common among female subjects and subtype 3 more common among infected males. Our finding of female bias with ST1 agrees with a study in Libya where *Blastocystis* ST1 infection was also significantly associated with females but was linked also to a low educational level [19]. Nevertheless, female sex-bias has not been detected in all studies. No significant associations between sex and subtypes of *Blastocystis* were found in Turkey [33, 34, 38]. The reasons for these marked differences in the relative proportions of subtypes between the sexes in affected populations are not currently understood, but they most likely relate to differences between the sexes in cultural/traditional patterns of behaviour and hence to different exposure rates to the sources of transmission [39].

## Conclusions

On the basis of our results, we recommend that stool screening via microscopy for the presence of *Blastocystis* should be largely abandoned since it is extremely insensitive even in the hands of the most experienced technicians. In future, the detection of *Blastocystis* infections should be based on PCR methodology and we predict that in the years ahead diagnostic PCR will become the tool of choice [40]. Our results have raised a number of interesting issues that will be addressed in future work, notably the underlying reasons for the regional trends in the prevalence of the three STs of *Blastocystis* that we detected in our study population, and for the sex bias in the prevalence of ST1 and ST3.
